# Intra‐abdominal omental mass as a desmoplastic round cell tumor: A rare case report

**DOI:** 10.1002/ccr3.8191

**Published:** 2023-11-15

**Authors:** Sujan Bohara, Pinky Jha, Pawan Singh Bhat, Srijan Malla, Samikshya Karki, Saroj Kumar Jha, Sunil Basukala, Sushil Bahadur Rawal

**Affiliations:** ^1^ Department of General and Gastrointestinal Surgery Nepal Mediciti Lalitpur Nepal; ^2^ Department of Surgery Shree Birendra Hospital Kathmandu Nepal; ^3^ Department of Physical Medicine and Rehabilitation Spinal Injury Rehabilitation Center Sanga Nepal; ^4^ Department of Emergency Medicine Gajendra Narayan Singh Hospital Rajbiraj Nepal

**Keywords:** case report, desmoplastic round cell tumor, mesenchymal, omentum

## Abstract

**Key Clinical Message:**

Desmoplastic round cell tumor, though rare, must be taken into consideration as a differential diagnosis, thus aiding in early evaluation and changing the trajectory of the natural history of the disease condition, and improving the prognosis of patients.

**Abstract:**

Desmoplastic small round cell tumor is a rare, aggressive tumor of mesenchymal origin with an incidence of 0.74 cases per million. We present a young adult with a periumbilical mass who was diagnosed as a desmoplastic round cell tumor and later was treated with exploratory laparotomy and resection of the tumor with no recurrence during a 6‐month follow‐up period.

## Graphical abstract

Histopathological image of desmoplastic small round cell tumor.

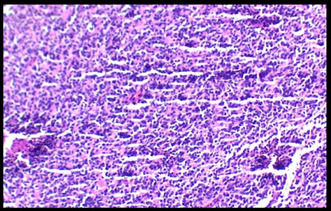

## 1 INTRODUCTION

Desmoplastic small round cell tumor is an extremely rare, aggressive mesenchymal tumor first described by Gerald et al. in 1898.^1^, ^2^ DSRCT is histologically distinguished by solid clusters of undifferentiated small round cells admixed in the dense desmoplastic stroma, and they most commonly manifest as multiple masses in the abdominopelvic cavity with no apparent origin, growing on the peritoneal surface of the serosal lining.^1^, ^2^, ^3^, ^4^ With a male‐to‐female ratio of 4:1 and a peak incidence of 0.74 cases per million, it primarily affects children and young adolescent populations.^5^

Contrast‐enhanced computed tomography (CECT) scan is widely used for the initial diagnostic imaging modality, followed by core‐needle biopsy and immunohistochemical analysis for epithelial, mesenchymal, and neural markers (cytokeratin, desmin, and vimentin), and neuron‐specific enolase. Since there is no established standard management protocol for the care of DSCRT, treatment modalities entail cytoreductive surgical debulking, high‐dose alkylator‐based chemotherapy regimens, or immunotherapy as a possible alternative treatment modality.^2^, ^5^

We present a young adult with a desmoplastic small round cell tumor who presented with a periumbilical mass and was treated with exploratory laparotomy and resection of the tumor with no significant recurrence during a 6‐month follow‐up period.

## 2 CASE PRESENTATION

A 25‐year‐old male presented to our center for the evaluation of a periumbilical mass for 5 months, which was insidious at the onset, gradually increasing with a rapid increase in the last 2 months, and associated with mild dragging‐type pain for the last month. He was initially addressed at the local level with the diagnosis of gastritis, but in contrast, this was not resolved. Thereafter, he was under traditional treatment for the last month. Furthermore, he provided a history of progressive weight loss of approximately 4 kg in the last month. The patient denied any history of serious medical conditions in the past or a significant family history of cancer. He was a non‐smoker and non‐alcoholic.

An abdominal examination revealed a firm, smooth‐surfaced, intraperitoneal, mobile mass that measured approximately 18 × 15 cm in the umbilical, hypogastrium, right lumbar, and iliac regions. However, other systemic examinations revealed no significant abnormalities.

**FIGURE 1:**
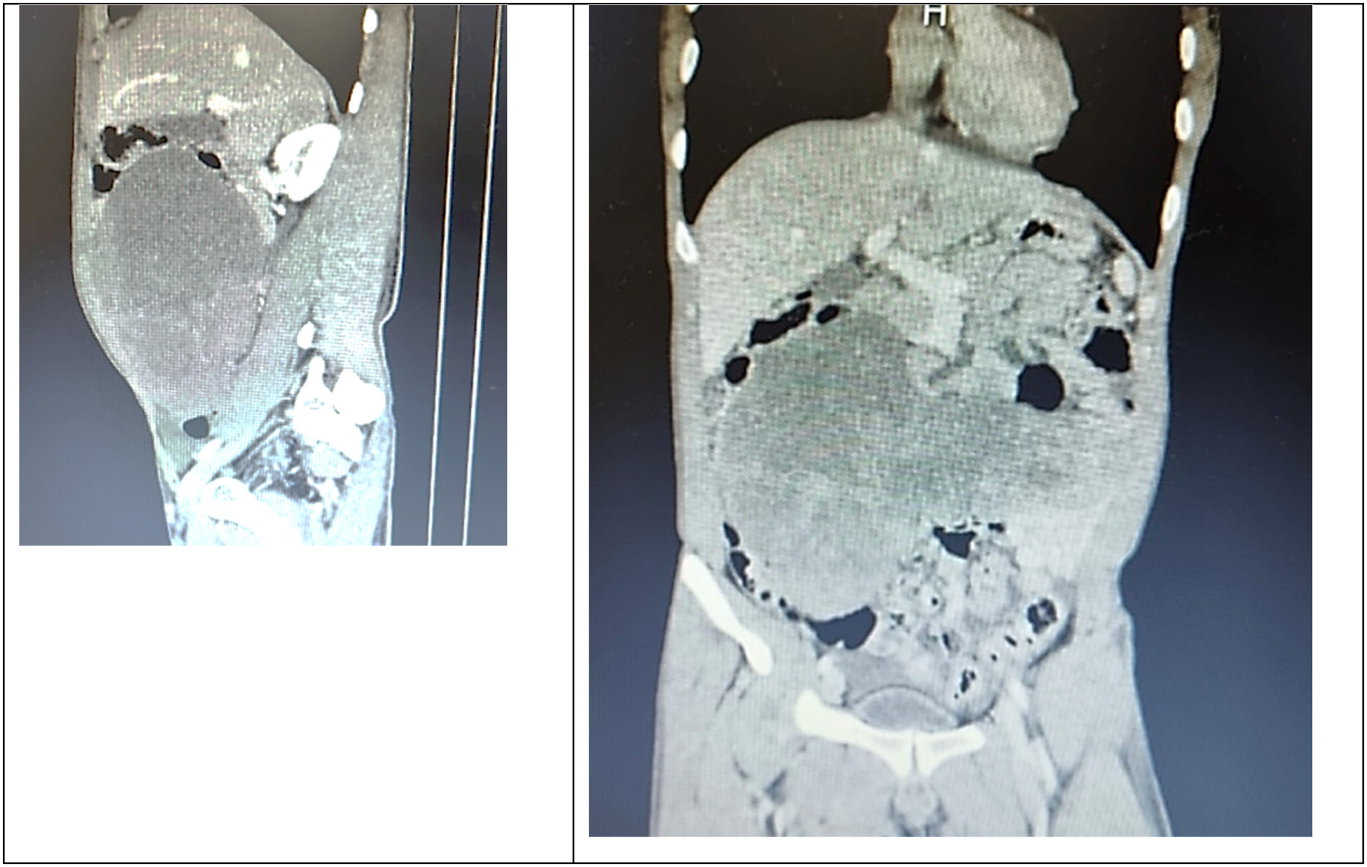
Contrast‐enhanced computed tomography images show huge soft tissue attenuation, heterogeneously enhancing intraperitoneal mass in the right mid, central, left mid, and right lower abdomen.

The CECT abdomen (Figure 1) revealed a huge soft tissue attenuation, heterogeneously enhancing, 21 × 17 × 11.4 cm intraperitoneal mass in the right mid, central, left mid, and right lower abdomen. The mass showed scattered foci of calcification. Multiple non‐enhancing hypodense cystic and necrotic areas were noted within this mass. Mesenteric vessels were displaced posteriorly, whereas small bowel loops were displaced peripherally by the mass. A small amount of free fluid was present in the pelvis.

**FIGURE 2:**
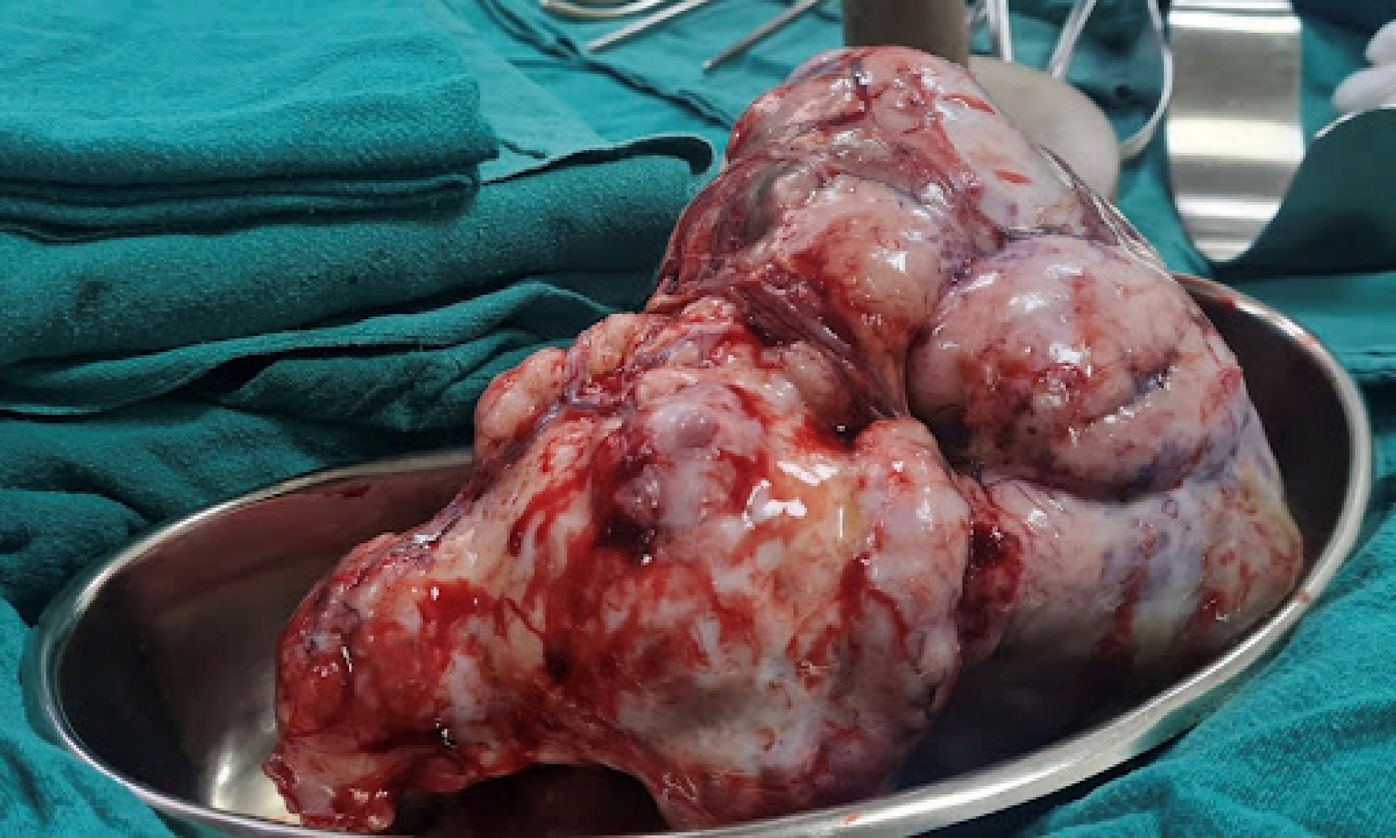
Gross picture of a large mass arising from the omentum, pedunculated with flimsy adhesion to the peritoneum of the right anterior abdominal wall.

**FIGURE 3:**
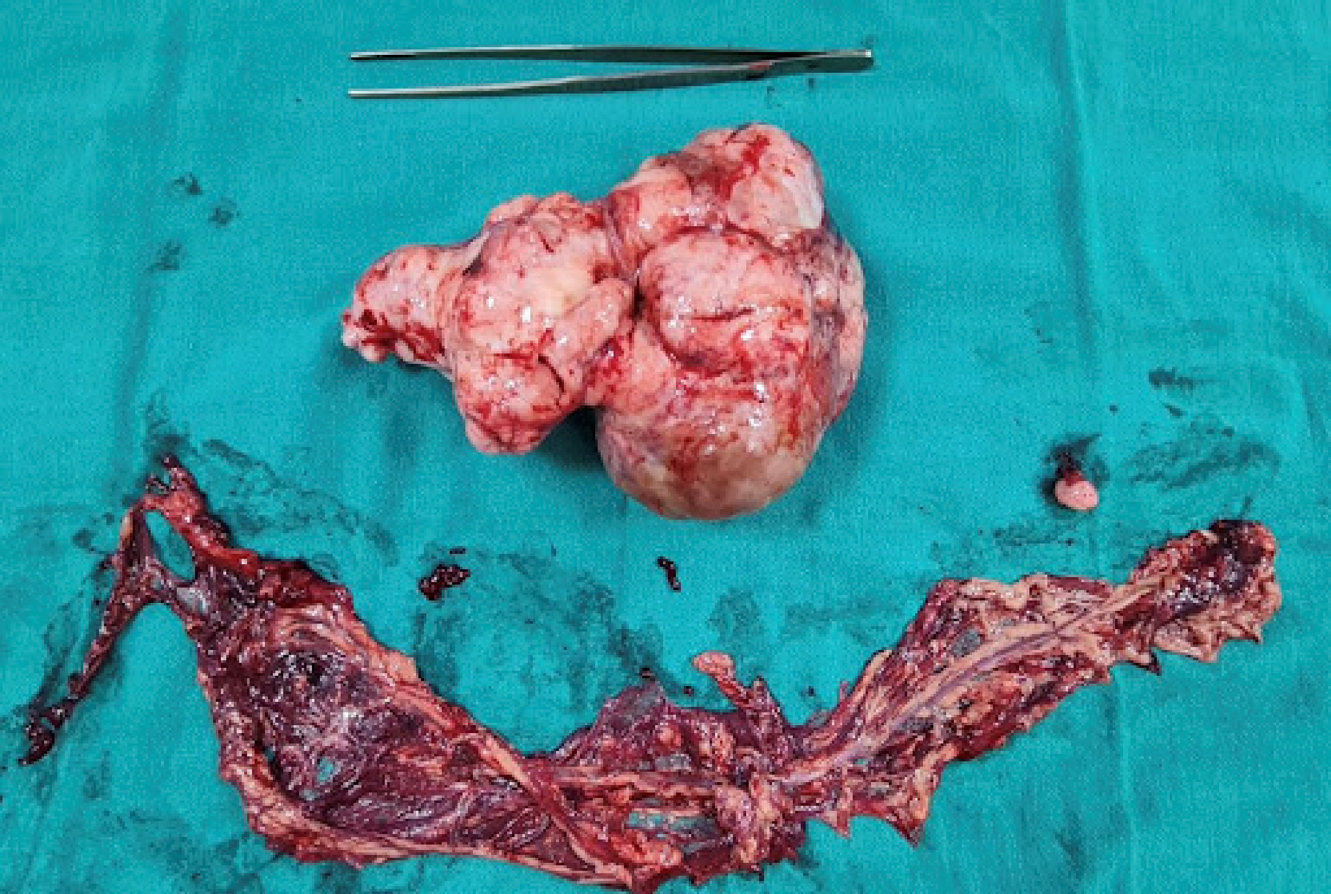
Gross picture of smaller masses arising from the omentum separately (in comparison with the size of large mass).

Based on these findings, exploratory laparotomy with excision of omental mass plus total omentectomy along with right‐sided peritonectomy under general anesthesia was performed. A large mass (22 × 18 × 15 cm; Figure 2) arising from the omentum, pedunculated with flimsy adhesion to the peritoneum of the right anterior abdominal wall, was discovered intraoperatively. Smaller masses (2 × 2 × 2 cm; Figure 3) arising from the omentum separately with minimal ascites were present.

**FIGURE 4:**
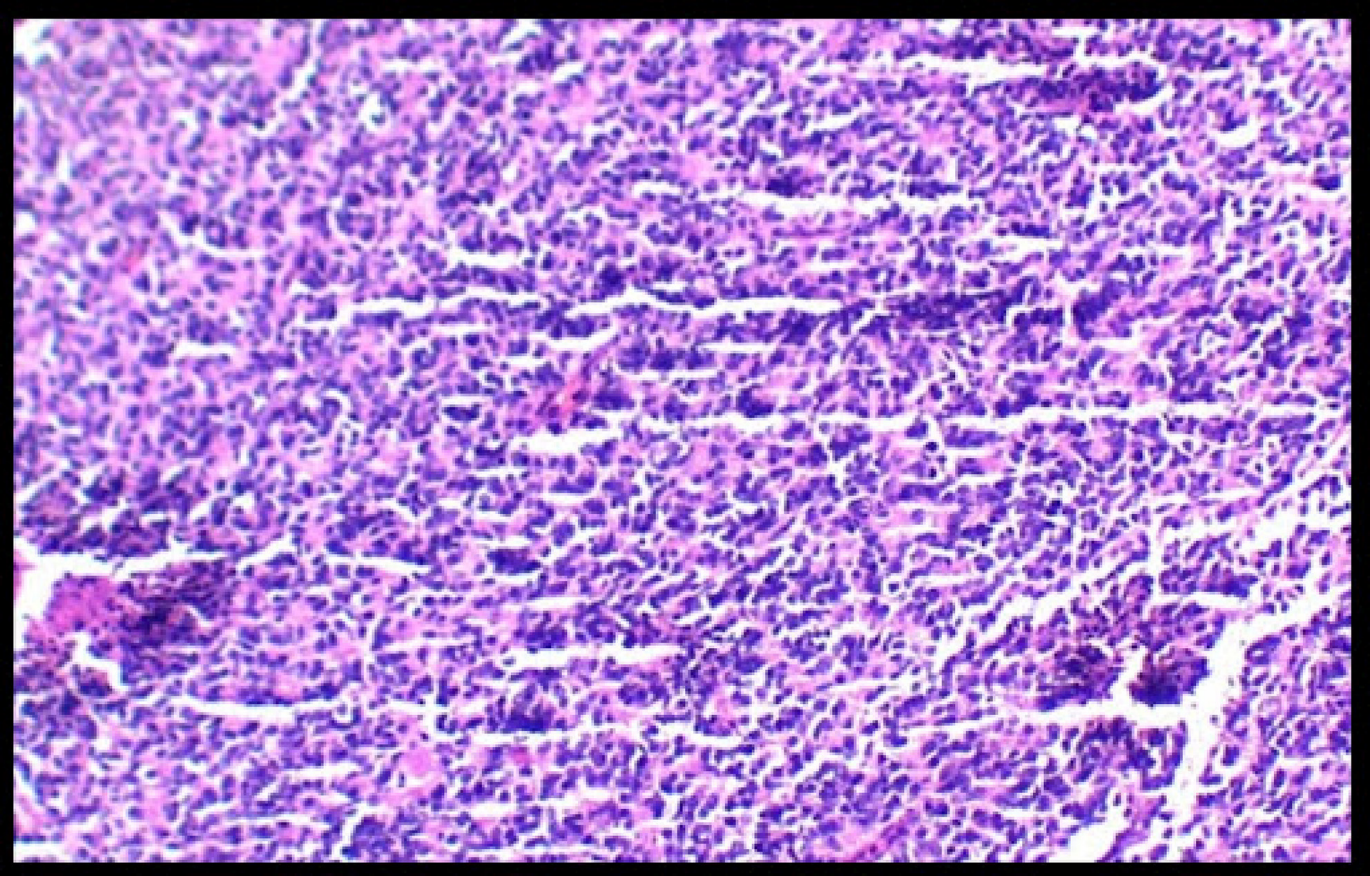
Histopathological specimen of omentum suggestive of desmoplastic small round cell tumor.

Histopathological examination (HPE) of the omental mass (Figure 4) revealed a surgical (capsular) margin involved by the tumor along with lymphovascular invasion and a single lymph node that is free of the tumor, as well as HPE of the peritoneum, which revealed no tumor cell infiltration, thus morphologically suggesting a desmoplastic small round cell tumor.

**FIGURE 5:**
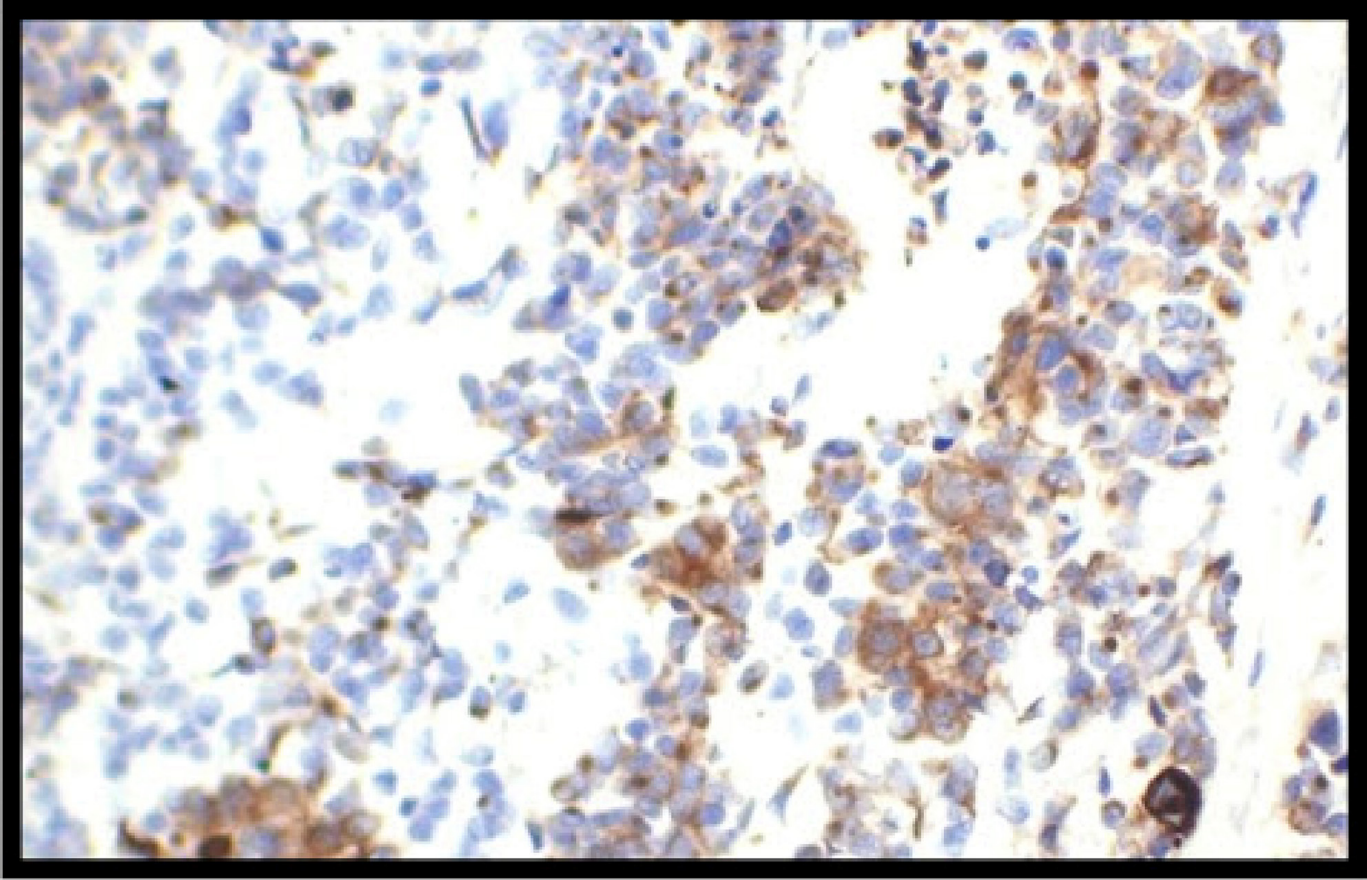
Immunohistochemical image of PanCK (AE1/AE3)‐ positive status.

**FIGURE 6:**
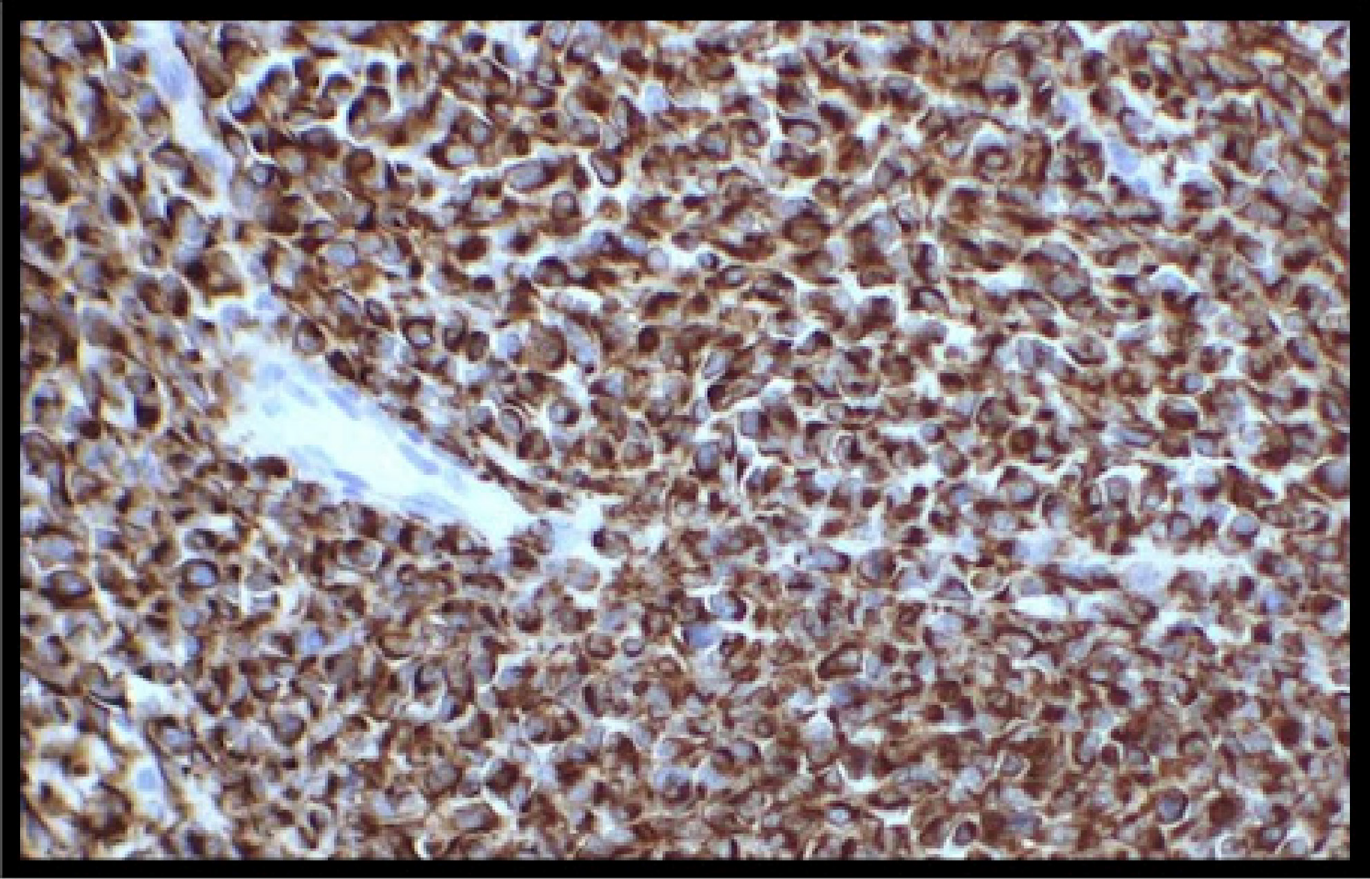
Immunohistochemical image of Desmin (GM 007)‐ positive status.

Furthermore, the immunohistochemistry (IHC) customized panel; WT1, EMA, Desmin, CK, Myogenin, SMA, Vimentin, CD99, NKX2.2, Chromogranin, CD45, and Ki‐67, as well as FISH to assess for EWSR1‐WT, were done, among which PanCK (AE1/AE3) and Desmin (GM 007) showed positive status (Figures 5 and 6), respectively. Thus, these findings were suggestive of a desmoplastic small round cell tumor. The postoperative period was uneventful and was discharged on the fifth postoperative day (POD), whereas suture removal was done on the 14th POD. Follow‐up after 3 and 6 months failed to show any recurrence.

## 3 DISCUSSION

Desmoplastic small round cell tumor is a rare high‐grade malignant tumor of mesenchymal cell origin.^6^, ^7^ As in our case, it is more commonly seen in the young male population, usually with an average age being 22 years old.^8^, ^9^ The long‐term prognosis remains poor. This usually occurs in the abdominal cavity, known as the intra‐abdominal desmoplastic small round cell tumor (IDSRCT). Other sites include the kidneys, bladder, lungs, pancreas, sinuses, and parotid glands.^2^ Tumors are most common on omental, mesenteric, or pelvic peritoneal surfaces.^6^

Gerald and Rosai describe the majority of IDSRCT's genetic makeup as a chromosomal translocation t (11; 22) (p13; q12) that resulted from the fusion of the Ewing's sarcoma gene's (EWS) N‐ and C‐terminal domains with the Wilms tumor suppressor gene's (WT1), creating the EWSR1‐WT1 gene.^2^, ^3^ Pathogenesis of this soft tissue sarcoma involves chromosomal translocation t (11; 22) (p13; q12) leading to fusion of the EWS and WT1 genes.^2^, ^8^ The fusion results in Ewing sarcoma RNA‐binding protein‐1. It typically manifests as a palpable abdominal mass accompanied by discomfort, ascites, jaundice, and nausea. Frequently, pressure symptoms are present such as hydronephrosis and constipation with or without weight loss.^6^

IDSRCT is frequently detected in a late stage. At the time of diagnosis, there may be even enlarged lymph nodes and distant metastases.^2^ A lobulated hypoechoic heterogeneous mass with enhanced vascularity and regions of hemorrhage, necrosis, and calcification is shown on ultrasound findings.^10^ Multiple peritoneal, omental, and serosal soft tissue masses are seen in the computed tomography (CT) results. Larger masses have low central attenuation with heterogeneous enhancement, whereas small masses demonstrate homogeneous low attenuation with uniform enhancement in CECT. Calcification frequently has a fine‐ to coarse‐speckled look.^11^ In our case, CECT imaging shows huge soft tissue attenuation, heterogeneously enhancing intraperitoneal mass. Magnetic resonance imaging (MRI) can find lesions that a CT scan cannot. Positron emission tomography (PET) imaging shows metabolic activity within tumor masses and is used for staging.^7^

The diagnostic test, fluorescent in situ hybridization (FISH), shows chromosomal translocation t (11; 22) (p13; q12), which causes the EWS and WT1 genes to fuse.^12^ Cytokeratin CAM5.2, desmin, keratin, WT1, vimentin, and neuron‐specific enolase are some of the positive histochemical markers.^9^, ^13^ Some cancers have epithelial membrane antigens CD99, FL1, and WT‐1 positive staining. On gross inspection, the tumor looks like a nonuniform white‐gray multinodular structure with histological finding of a nest of small round blue cells with scant cytoplasm and inconspicuous nucleoli, embedded in desmoplastic fibrous connective tissue.^2^, ^11^, ^14^ Immunohistochemistry and translocation studies are crucial to differentiate DSRCT from Ewing sarcoma and other sarcomas that have similar appearances.^15^ The differential diagnosis includes malignancies such as neuroblastoma, rhabdomyosarcoma, PNET and peritoneal leiomyosarcomatosis, lymphoma, and intra‐abdominal desmoid tumor.^7^ Some granulomatous infections as tuberculosis can mimic the condition as well.^10^

For the treatment of DSCRT, multimodal therapy is being considered. The disease can be controlled by combining high‐dose chemotherapy with extensive surgery and tumor debulking. Irradiating the entire abdomen may marginally increase long‐term survival.^8^ The P6 protocol, also known as the VAC‐IE regimen, contains the greatest alkylating agents, was successful, according to a study by Jayakrishnan et al.^9^ Adjuvant therapy comprises radiotherapy, either with or without chemotherapy. Radiotherapy does not improve overall survival however improves the outcome of the patients. Prognosis is relatively poor with a 3‐year survival rate of 50% in aggressively treated with surgical resection.^7^ Recurrence is very common in this condition causing long‐term survival has been reported to be only 8%.^16^ Our patient during follow‐up on the third and sixth months did not show recurrence.

## 4 CONCLUSION

DSRCT must be taken into consideration while making a differential diagnosis for young individuals with abdominal tumors. Early diagnosis can help in improving the prognosis of the condition.

## AUTHOR CONTRIBUTIONS

**Sujan Bohara:** Conceptualization; resources; writing – original draft; writing – review and editing. **Pinky Jha:** Conceptualization; resources; writing – original draft; writing – review and editing. **Pawan Singh Bhat:** Data curation; supervision; writing – review and editing. **Srijan Malla:** Data curation; supervision; writing – review and editing. **Samikshya Karki:** Resources; writing – review and editing. **Saroj Kumar Jha:** Resources; writing – review and editing. **Sunil Basukala:** Supervision; writing – review and editing. **Sushil Bahadur Rawal:** Supervision; writing – review and editing.

## FUNDING INFORMATION

All authors declare that they have not received any grants or funding for this manuscript.

## CONFLICT OF INTEREST STATEMENT

All the authors declare that they have no conflicts of interest.

## ETHICS STATEMENT

Not required for the publication of this manuscript.

## CONSENT

Written informed consent was obtained from the patient to publish this report in accordance with the journal's patient consent policy.

The Abstract has been submitted to the 5th International Cancer Congress held on March 17–18, 2023, in Janakpurdham, Nepal.

## Data Availability

The data used to support the findings of this study are available from the corresponding author upon request.
